# Modern Sociology

**Published:** 1903-08-08

**Authors:** 


### Modern Sociology.

### PUBLIC HEALTH IN SCOTLAND.

In a recent number of *The British Medical Journal* there is an address read before the Medico-Chirurgical Society of Edinburgh by Dr. Clouston, in which he proceeds to examine the national conscience, and especially the consciences of the Scottish medical corporations, in reference to the ignorance of the population in general sanitary matters. The sanitary condition of Scottish palaces revealed by recent Parliamentary questions might perhaps lead one to infer that this ignorance of the conditions of health was voluntary in the higher classes, while the masses might be supposed to exist under conditions in which the limit of sanitary progress was typified by the works of the good Duke of Argyll. This, however, is not the case. As might be expected from a population in which the general diffusion of education and instruction has always been greater than in any of the other parts of the United Kingdom, the process of sanitary education has long been going on. "The conviction of health-sin, is general, and the prospects of conversion to a new and healthy life are promising." Health and sanitary inspectors, notification of infectious diseases, inspection of workshops, health lectures, health congresses innumerable, and energetic efforts by medical societies and by medical and lay preachers of physical righteousness have all, in addition to prolonging life and diminishing death, done incalculable service by causing in the public mind "a dissipation of the former fatalistic assumption that disease and deformity, weakness and unfitness, are inevitable things which God and the devil had arranged between them for the chastening of mankind and the punishment of their sins." A health conscience is being slowly created. Many people are keenly dissatisfied with the present health and welfare of the body politic and self-reproachful about evils which they think should not exist, and which they individually should help to put an end to." Notwithstanding all this, Dr. Clouston says the public ignorance as to health and welfare conditions is great, but the craving to know them is widespread and pathetic. A professional authority to which appeal can be made is longed for, and he proceeds to inquire in how far the Scottish Medical Corporations and the profession in Scotland have taken the position as teachers to which their knowledge of physiological and sanitary laws entitles them. Has the profession taken adequate steps to place its knowledge of accurate chemical and physiological facts regarding food "that primary of all physiological needs" in a useful and easily comprehensible form before the people? On this point Dr. Clouston refers to "A Study of the Diet of the Labouring Classes in Edinburgh" by Drs. Noel Paton, Crauford Dunlop, and E. M. Inglis, published last year, and which in part suggested to him the address at present under consideration. This work embodied most laborious and careful observations, it was intensely practical, and its facts and conclusions would, if digested and practised, have been of great benefit to every wage-earner in Scotland. "Life and health to many of them might depend on a knowledge of that paper." Yet no systematic means were taken to spread the knowledge so laboriously won, so carefully compiled, in an effective way amongst the people it concerned. "Our physiological, medical, sanitary and dietetic knowledge of the most pressingly practical value is buried in our medical journals and books and never reaches or benefits the people who need it and who often would be most willing to receive it." With regard to the relation of the important work of education to the public health, and especially of fitting the studies to the capacities of the scholars, he calls attention to the very insignificant place held by physiology and medicine in the preparation of the teachers, and illustrates it by a remark made by Professor Edgar when addressing a body of teachers "that educators were commonly ignorant of the very first principles of the physiology of the developing brain"—a remark which was not contradicted. He further refers to the awaking sense of the country to the idea that there is a process of physical degeneration going on in town populations and to the fact that there has been no large and scientific treatment of this momentous subject. Scientific men alone can treat this matter fully, but "at present we drift along in ignorance, with spasms of panic when such facts as that only one Manchester recruit out of five can be made to handle a rifle" come out in a casual way. It is urgently necessary to improve the health and organic welfare of the people, and recent advances in science and medicine render it possible to do so. Dr. Clouston's paper deals with the public health of Scotland, but his remarks have an universal application. "Our people are on the whole well educated and receptive, as well as being alive to their own interests. Our newspaper press is public spirited, and, when politics do not intervene, open to all who can interest and instruct their readers. We have scattered through the country thousands of medical men increasingly well instructed, many of them being zealous and not too busy. We have in our corporations an increasing number of young Fellows and Members, with all kinds of scientific knowledge at command, eager for research and for work of every sort." He proposes that the medical corporations should appoint a large and representative joint health committee with powers of inquiry and suggestion, which on account of the importance of its work to the people should be financed by the State. To this body all questions affecting the public would be referred and the best means of dealing with them determined. Dr. Clouston draws a somewhat roseate picture of the benefits that would accrue to the population from the authoritative teaching furnished by the collective wisdom of this committee. "Pregnancy and parturition would be bereft of many of their gloomy forebodings; lactation, child-feeding and nurture would be based on scientific knowledge, new milk and pure milk only would be given to children amongst whom the mortality would fall 50 per cent. in our cities. . . . The effects of good air and bad air breathed by children would be appreciated. . . . The teacher would have far more physiological knowledge of the human brain and human body than he has now. The distinctions between physiological and moral sins would be made at home and at school. . . . The real meaning and the risks of puberty and adolescence would be far better understood. . . . Moral and religious training would go hand in hand with health training and would be considered a part of it. . . . The all-important marriage question would have some science and reason as well as emotion come into it. . . . Deliberately to choose to live in a crowded or unhealthy neighbourhood would be regarded as suicide is now. To use alcohol in excess, to indulge in sexual vice would be equivalent to losing self-respect and manliness. Health would be idealised and longed for as beauty is in woman. . . . Disease and death would be more painless than they commonly are. Life would be far more efficient than it is now." Whether all these results are likely to be attained in such perfection as Dr. Clouston anticipates by the means he suggests is of course open to argument, but there can be no doubt of the soundness of the ground he takes in declaring that in these as in other matters affecting national weal progress can be made only by the education and instruction of the people.

